# A new congenital multicore titinopathy associated with fast myosin heavy chain deficiency

**DOI:** 10.1002/acn3.51031

**Published:** 2020-04-19

**Authors:** Aurélien Perrin, Corinne Metay, Marcello Villanova, Robert‐Yves Carlier, Elena Pegoraro, Raul Juntas Morales, Tanya Stojkovic, Isabelle Richard, Pascale Richard, Norma B. Romero, Henk Granzier, Michel Koenig, Edoardo Malfatti, Mireille Cossée

**Affiliations:** ^1^ Laboratoire de Génétique Moléculaire CHU de Montpellier Montpellier France; ^2^ Laboratoire de Génétique Moléculaire de Maladies Rares EA 7402 Université de Montpellier Montpellier France; ^3^ Unité Fonctionnelle de Cardiogénétique et Myogénétique Moléculaire et Cellulaire Centre de Génétique Moléculaire et Chromosomique et INSERM UMRS 974 Institut de Myologie Groupe Hospitalier La Pitié‐Salpêtrière‐Charles Foix, Paris INSERM UMRS1166 UPMC Paris 6 Paris France; ^4^ Neuromuscular Rehabilitation Unit Nirgisoli Hopsital Bologna Italy; ^5^ DMU Smart Imaging Medical Imaging Department Raymond Poincaré Teaching Hospital Assistance Publique des Hôpitaux de Paris (AP‐HP) GHU Paris‐Saclay University Garches France; ^6^ INSERM U 1179 University of Versailles Saint‐Quentin‐en‐Yvelines (UVSQ) Paris‐Saclay Garches France; ^7^ Neurology Department Padova University Padova Italy; ^8^ Myology Institute Neuromuscular Pathology Reference Center Groupe Hospitalier Universitaire La Pitié‐Salpêtrière Paris France; ^9^ Sorbonne Universités UPMC Univ Paris 06 Paris France; ^10^ Généthon INSERM UMR951 INTEGRARE Research Unit 91002 Evry France; ^11^ Unit of Neuromuscular Morphology Institute of Myology Pitié‐SalpêtrièreUniversity Hospital Paris France; ^12^ Department of Cellular and Molecular Medicine University of Arizona MRB 325. 1656 E Mabel Street Tucson Arizona 85724‐5217; ^13^ Service Neurologie Médicale Centre de Référence Maladies Neuromusculaires Nord‐Est‐Ile‐de‐France CHU Raymond‐Poincaré Garches France; ^14^ U1179 UVSQ‐INSERM Handicap Neuromusculaire: Physiologie, Biothérapie et Pharmacologie Appliquées UFR des Sciences de la Santé Simone Veil Université Versailles‐Saint‐Quentin‐en‐Yvelines Versailles France

## Abstract

Congenital titinopathies are myopathies with variable phenotypes and inheritance modes. Here, we fully characterized, using an integrated approach (deep phenotyping, muscle morphology, mRNA and protein evaluation in muscle biopsies), two siblings with congenital multicore myopathy harboring three *TTN* variants predicted to affect titin stability and titin‐myosin interactions. Muscle biopsies showed multicores*,* type 1 fiber uniformity and sarcomeric structure disruption with some thick filament loss. Immunohistochemistry and Western blotting revealed a marked reduction of fast myosin heavy chain isoforms. This is the first observation of a titinopathy suggesting that titin defect leads to secondary loss of fast myosin heavy chain isoforms.

## Introduction

Titin is the largest human protein and is encoded by the titin gene (*TTN*) (OMIM*188840). Titin is expressed in cardiac and skeletal muscles, and one molecule spans half the length of a sarcomere.^1^ Next‐generation sequencing (NGS) data indicate that *TTN* is a major causative gene in neuromuscular disorders.^2^ The term congenital titinopathy defines a group of disorders with axial‐predominant weakness, variable cardiac involvement,^3^ and heterogeneous histopathological features.^4^ We recently demonstrated that congenital titinopathies have a common ultrastructural signature characterized by focal clear areas of disorganization with M‐line dissolution, leading to loss of the sarcomeric scaffolding.^3^, ^5^ The mechanisms underlying titinopathy phenotypic variability, muscle weakness, and variable inheritance are poorly understood. They might involve direct alterations in titin structural functions, such as sarcomere formation and stability, and also abnormal interactions of titin with other proteins^6^ that might lead to secondary protein alterations. For example, it is well known that aberrations in titin MEX6 domain cause secondary calpain 3 deficiency and contractile myopathy.^7^ However, little is known about other possible protein alterations linked to titin deficiency.

From a genetic point of view, *TTN* variants may be silent or have a dominant or recessive effect. Bioinformatics tools lack predictive value for evaluating the pathogenicity of mutations, especially *TTN* missense variants. Moreover, *TTN* variants are very frequent in the general population.^2^ We recently developed a variant prioritization score called MoBiDiC prioritization algorithm (MPA).^8^ As MPA aggregates the results of several predictors, individual predictor errors are counterweighted, improving the sensitivity and specificity of pathogenicity predictions for missense and splice variants. The MPA score can efficiently prioritize the large number of *TTN* variants identified in patients. Moreover, TITINdb, a web application that integrates information on titin structure, sequence, isoforms, and variants,^9^ is interesting for predicting the potential effect of *TTN* missense variants. Considering the high number of *TTN* variants of uncertain significance, it is crucial to include mRNA and protein analyses when assessing the effects of *TTN* variants on titin transcripts, quantity, size, and functionality. It is now clear that clinical, morphological, genomic, mRNA, and protein data must be combined to reach a definite diagnosis.^5^

Here, we describe three *TTN* variants, one frameshift mutation (c.79683dupA; p.(Arg26562Thrfs*12)) and two missense mutations (p.(Thr31339Ala) and p.(Thr6324Pro)), in two siblings with congenital multicore myopathy. This family was previously included in the study by Savarese et al.^10^ who analyzed 93 neuromuscular genes and provided a workflow for interpreting *TTN* variants. In this report, we present additional phenotypical and molecular data that establish the pathogenicity of these *TTN* variants. We also detected the loss of the myosin heavy chain (MyHC) fast isoforms, suggesting that this titin deficiency leads to secondary loss of fast myosin heavy chain isoforms.

## Methods

This study was approved by the Ethics Committee and followed the ethical guidelines of our institutions for clinical studies in compliance with the Helsinki Declaration. Patients or parents signed the informed consent for the genetic analysis according to French legislation (Comité de Protection des Personnes Est IV DC‐2012‐1693).

### Morphological analysis

Muscle biopsies were obtained from P1 at the age of 7 years (vastus lateralis muscle) and of 18 years (deltoid muscle) and from his healthy parents at 48 and 58 years of age (deltoid muscle).

Standardized histochemical techniques and electron microscopy analyses were performed, as previously described.^11^ Immunofluorescence studies were done using antibodies against myosin alpha and beta (slow) heavy chain (6H1, Developmental Studies Hybridoma Bank, University of Iowa, Iowa City, IA), fast 2B heavy chain (BF‐F3, Developmental Studies Hybridoma Bank, University of Iowa). BA‐D5, and both fast 2A heavy chain, and type 2X‐MyHC (SC‐71, Developmental Studies Hybridoma Bank, University of Iowa).

### Next‐generation sequencing

NGS analysis to detect single‐nucleotide and copy‐number variants were performed as previously described,^12^ using a specific custom‐designed panel of 54 genes (Table S1). Paired‐end sequencing was performed on a 250 cycle Flow Cell (Illumina, Santa Cruz, CA) and the Illumina MiSeq platform.

### Variant interpretation

*TTN* variant pathogenicity was determined according to the current ACMG guidelines. The predicted effects on transcripts and translation were based on several criteria: frequency in the general population (GnomAD [http://gnomad.broadinstitute.org/]), alteration or not of the reading frame, the potential implication of functional domains, and bioinformatic predictions (MPA^8^ and TITINdb [http://fraternalilab.kcl.ac.uk/TITINdb/]). To take into account the complexity of skeletal *TTN* transcripts, particularly the fact that some exons are expressed only during fetal development (metatranscript‐only exons), the recent study of transcripts by Savarese et al^13^ was used as a reference for this analysis.

*TTN* variants were confirmed by Sanger sequencing.

### WB analysis

Western blot (WB) experiments were performed using deltoid muscle biopsies^14^ and antibodies against titin C‐terminal (rabbit M10‐1; 1:1000)^15^ and N‐terminal part (mouse; 1:1000; Sigma SAB1400284, St. Louis, MO), slow MyHC 1 (mouse; 1:3500; Sigma M8421) and fast MyHC2A and 2X (mouse; 1:4500; Sigma M4276). Experiments included also muscle biopsy specimens from a healthy control (from the Myobank‐AFM) and from patient i703 with the c.106139dupA, p.(Ser35381Glufs*4) homozygous C terminal frameshift variant (positive control).

### Transcripts analysis

To analyze the consequences of *TTN* variants on exon 326 skipping, mRNA was extracted from 20 mg of muscle biopsy and prepared as described in Ref. ^16^. Two *μ*g of RNA was reverse transcribed with Invitrogen Superscript II reverse transcriptase. Then, first‐strand DNA was amplified by PCR with the following primers: exon 326 forward 5′‐ACAGAAGTGGCATTCGATGGAT‐3′ and reverse 5′‐TGGACGACCTTTGAATGGAATG‐3′*.*

### Whole‐body MRI

For each patient, 350 5‐mm thick, contiguous sections were obtained by multi‐stack IDEAL T2 (DIXON) imaging. In each section (from head to toes), muscles were evaluated using in and out phase images and also water and fat images.

## RESULTS

### Clinical findings

The two siblings, an 18‐year‐old man (P1) and his 10‐year‐old sister (P2), were born to non‐consanguineous healthy Italian parents (summary in Table 1).

**Table 1:** Clinical characterization of the two patients.

	Patient P1	Patient P2
Disease onset	Antenatal	Neonatal
Motor milestones	Retarded	Retarded
Disease course	Slowly progressive. Never ran At 18 years: walk <500 m Wheelchair needed for outdoor	Slowly progressive At 11 years: normal walking perimeter Wheelchair occasionally needed
Distal weakness (MRC grade)	Weakness of finger flexors and extensors (4). Foot dorsiflexors (4)	Weakness of finger flexors and extensors (4). Loss of foot dorsiflexion (2)
Axial weakness (MRC grade)	Neck flexors (3)	Neck flexors (2)
Proximal weakness (MRC grade)	Upper limbs (4). Lower limbs (3)	Upper and lower limbs (3)
Facial weakness	Bilateral ptosis, facial weakness	Bilateral ptosis, facial weakness
Skin alterations	Severe hyperkeratosis pilaris	Mild ptosis, asymmetric facial diplegia
Bone deformities	Mild scoliosis, high‐arched palate, scapular winging, pectus excavatum, genu valgum	High‐arched palate, lumbar hyperlordosis, scapular winging
Joint alterations	Hyperlaxity, Achilles tendon contracture	Hyperlaxity, left Achilles tendon contracture
EMG	Myopathic	Myopathic
Lung function	Recurrent respiratory infections, FVC 58%	FVC 60%
Cardiac involvement	Right branch block, mitral valve prolapse Holter ECG normal	Bradycardia at birth. Bradycardia with syncopal episodes, slight mitral valve prolapse with mild valve insufficiency
Creatine kinase level	Normal	Normal

EMG, electromyography; MRC, Medical Research Council scale for muscle strength; FVC, forced vital capacity.

**Figure 1:**
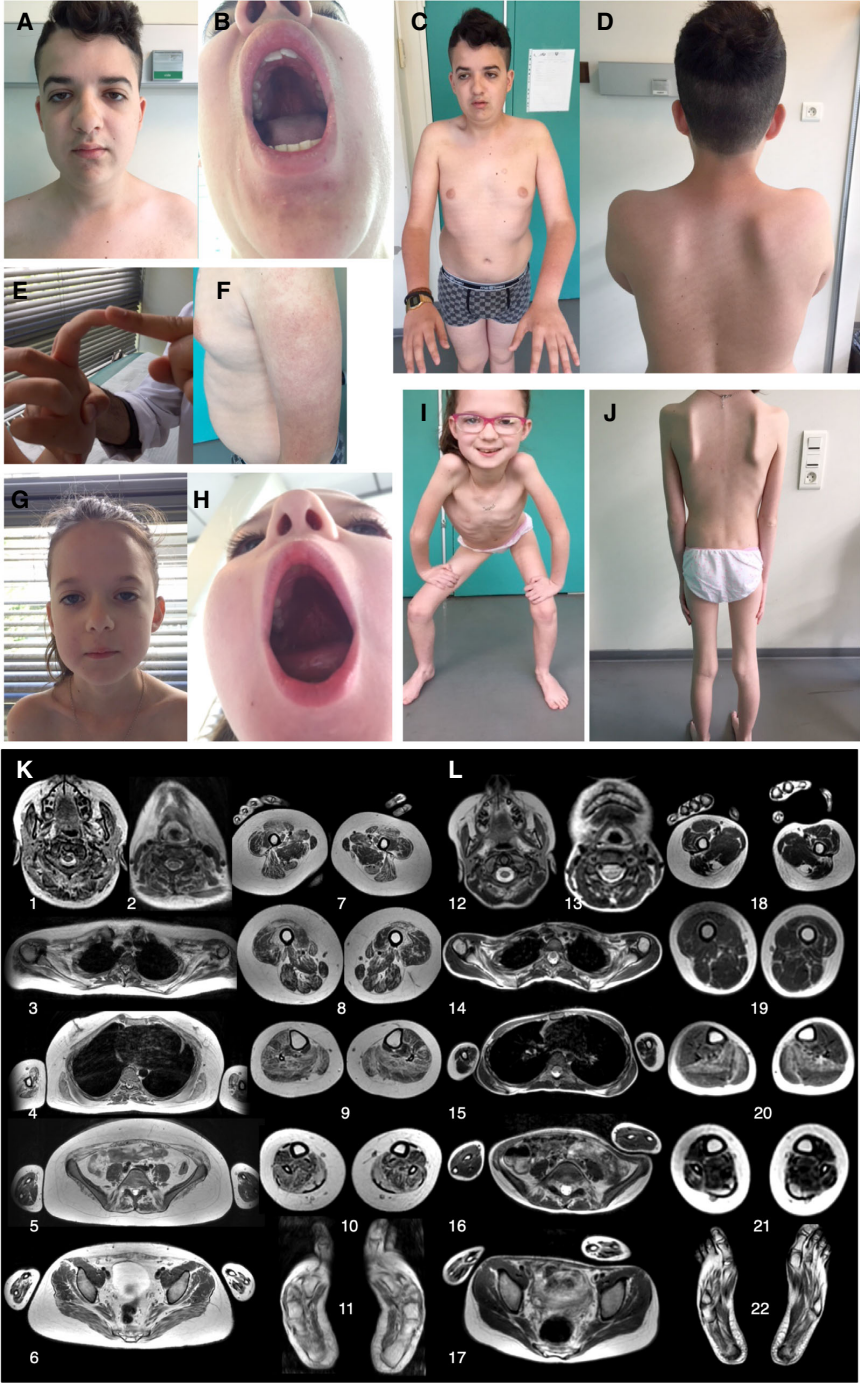
Clinical presentation and MRI features. (A–F) P1’s clinical features. (A) Mildly elongated face with bilateral ptosis and low‐set ears. (B) High‐arched palate. (C) Deficit in arm elevation (45°). (D) Scapular winging. (E) Distal joint hyperlaxity. (F) Prominent hyperkeratosis pilaris. (G–J) P2’s clinical features. (G) Mild ptosis without other facial dysmorphisms. (H) High‐arched palate. (I) Pelvic muscle proximal weakness; the patient cannot squat. Note the extremely thin muscle bulk. (J) Thin muscle bulk, mild scapular winging. (K and L) Three‐dimensional volume rendering reconstruction of MRI sequences in P1 (K1‐11) and P2 (L12‐22) with anterior, lateral and posterior views. These views allow a global analysis of the patients’ phenotype. Selection of 11 slices among the 350 mm thick, contiguous axial slices from head to toe; DIXON T2 (IDEAL T2) in‐phase images. P1 is older and the disease is more advanced with more fat infiltration and atrophy compared with P2. The distribution of involved muscles is similar but less pronounced in P2. Only one muscle is more fatty ‐infiltrated in P2. The upper part of the semi‐tendinous muscle is as white as subcutaneous fat in P1 (K‐7). The sternocleidomastoid, ileo‐psoas, *gracilis*, *adductor magnus* and common toe extensors are less affected by the disease.

P1 had a history of reduced fetal movements, major hypotonia, torticollis, strabismus and bilateral clubfoot at birth. From the age of 5 years, he manifested slowly progressive limb‐girdle and axial weakness with Achilles tendon contractures and joint hyperlaxity. He also presented bilateral ptosis, low‐set ears, and high‐arched palate (Fig. 1A and B). He also had a hyperkeratosis pilaris on his arms (Fig. 1F). Forced vital capacity (FVC) was 58%. ECG revealed a right branch block, and heart ultrasound showed mitral valve prolapse. Whole‐body muscle MRI showed diffuse and symmetric muscle involvement with preservation of the sternocleidomastoid, psoas, iliac, *gracilis*, *adductor magnus*, and toe extensors (Fig. 1K).

P2 had bradycardia during delivery. She presented failure to thrive, prominent axial weakness, head lag, and motor delay. She developed slowly progressive lower limb‐girdle muscle weakness from the age of 1.5 years and joint hyperlaxity. She had mild ptosis, high‐arched palate with limited mouth opening, small muscle bulk (Fig. 1G and H), and low body mass index (9.6 kg/m^2^; normal range: >18.5 to <24.9). Left Achilles tendon contractures were also observed. FVC was 60%. The cardiac work‐up revealed rhythm alterations with bradycardia and repetitive syncopal episodes. Heart ultrasound showed a slight mitral valve prolapse with mild valve insufficiency. Left ventricular ejection fraction was 63%. Heart MRI did not show cardiomyopathy. Whole‐body muscle MRI showed diffuse and symmetric muscle involvement, particularly the upper part of the semitendinosus muscles (Fig. 1L). Neurologic and cardiac exam of the parents was normal.

### Muscle morphological analysis

**Figure 2:**
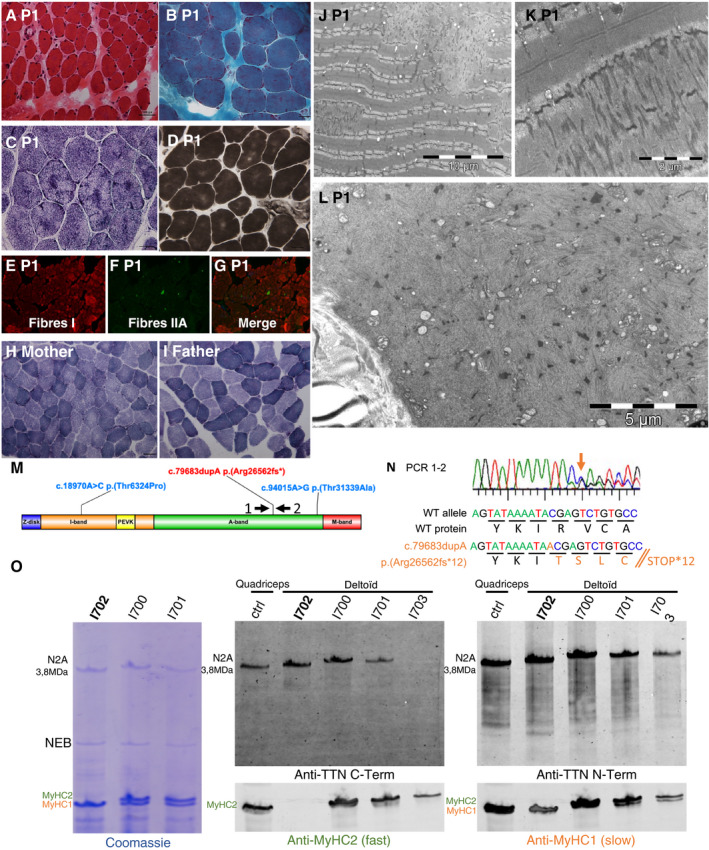
Skeletal muscle histology, genetic, transcripts and proteins analyses. (A–G) P1 deltoid muscle biopsy. (A) Hematoxylineosin staining. Presence of marked fiber size variation, nuclear internalization, and increased endomysial connective tissue. (B) Gömöri trichrome staining. Altered distribution of the mitochondrial network. (C) NADH. Multiple small areas of reduced oxidative activity alternating with areas of intense activity, conferring a blurred lobulated aspect to the fibers. Intense areas of oxidative staining are evident in some fibers. (D) ATPase pH 4.63. Absence of differentiation between type 1 and type 2 fibers. Only one color is detected. (E–G) Immunofluorescence analysis with antibodies against myosin alpha and beta‐slow heavy chain (E), fast 2A and 2X heavy chain (F), and merge. Presence of few scattered green fibers, demonstrating the almost complete absence of type 2 fibers that express fast myosin heavy chain isoforms. The merged image confirms this observation (G). Magnification 16×. (H) Deltoid muscle biopsy from the mother. NADH. Normal oxidative reaction. (I) Deltoid muscle biopsy from the father. NADH. Normal oxidative reaction. (J–L) Ultrastructural studies. (J) Focal and clear areas of sarcomeric loss corresponding to cores and disorganization with Z material accumulation. (K) Dense material originating from Z‐line accumulation along few sarcomeres. (L) A muscle fiber with completely disrupted sarcomeric structure and prominent myosin loss. (M) Localization of the titin variants identified by NGS in titin domain structure. (N) Transcript characterization and strategy used to analyze the transcript region containing exon 326. Primer 1 and 2 (see [M] for location) allowed confirming the presence of the mutation (orange arrow). However, the abundance of the mutated transcripts seemed to be reduced compared with wild type. (O) WB analysis (1% SDS‐agarose) of muscle biopsy protein lysates from P1 (i702) and his parents (i700 and i701) using the Odyssey^®^ protocol for antibodies incubation and detection. Ctrl: control muscle biopsy from a healthy individual (biopsy from Myobank‐AFM). i703: muscle biopsy from a patient with the homozygous variant c.106139dupA, p.(Ser35381Glufs*4) resulting in a C‐terminal truncated titin protein. No band was detected with the antibody against titin C‐terminus (M10.1), whereas a band was detected with the antibody against the N‐terminus (Sigma SAB 1400284). The small size reduction could not be observed due to lack of resolution. In P1 (i702), anti‐titin antibodies did not show any abnormality, particularly not the band of about 2600 kDa resulting from the frameshift variant in exon 326 (c.79683dupA). Coomassie blue staining showed loss of the band corresponding to the fast myosin heavy chain isoforms that was confirmed by WB with a fast MyHC2A and 2X antibody. No alteration in MyHC2 expression was observed in the parents’ biopsies.

Analysis of the deltoid muscle biopsy from P1 revealed marked fiber size variation, and increased endomysial and perimysial connective tissue (Fig. 2A). Gömöri trichrome staining showed an altered distribution of the mitochondrial network (Fig. 2B). Multiple areas of reduced oxidative activity, corresponding to multicores, alternating with areas of intense activity were present (Fig. 2C). In some fibers, there were central areas of intense oxidative reaction, resembling *“inverted central cores”* (Fig. 2C). ATPases showed the absence of differentiation (Fig. 2D). Immunofluorescence analysis highlighted the absence of fibers that express myosin 2B and the almost complete absence of fibers that express fast myosin 2A and 2X (Fig. 2E–G). The parents’ muscle biopsies did not show any significant alteration, and normal oxidative activity (Fig. 2H and I).

Ultrastructural studies of P1 biopsy revealed a loss of sarcomeric scaffolding, multiple cores with focal myofibrillar disorganization, dense Z‐line material, and absence of mitochondria corresponding to lesions observed with oxidative staining (Fig. 2J). In other fibers, the sarcomeric structure was in complete disarray (Fig. 2K). Myosin filaments had disappeared in the central areas of the lesions, and only small Z‐line fragments with thin filaments and some vesicles (Fig. 2L) were recognizable.

### *TTN* variants

NGS allowed identifying in both patients a frameshift *TTN* mutation in exon 326 (NM_001267550.1) c.79683dupA; p.(Arg26562Thrfs*12), inherited from the mother, and two missense mutations in exon 339: c.94015A>G; p.(Thr31339Ala) and in exon 65: c.18970A>C; p.(Thr6324Pro), inherited from the father.

The c.79683dupA variant is located in the A band and is predicted to lead to a truncated protein missing part of the A band and the myosin interaction domain.^6^ It has never been reported before in patients (Pubmed, LOVD and HGMD Pro) nor in the general population (GnomAD). The missense variants p.(Thr6324Pro) and p.(Thr31339Ala) are localized in the I‐band and A‐band domains, respectively. Thr31339 is in a myosin‐binding Fn3 domain, and its replacement by an Ala might affect binding to myosin and muscle contraction^17^ (Fig. 2M). The two missense variants have never been described (Pubmed, LOVD and HGMD Pro) and their allelic frequencies are unknown (GnomAD). The MPA in silico prediction tool suggested that the two missense variants are weakly pathogenic (score: 3/10 for both). TITINdb predicted a destabilizing effect.

No other pathogenic variant was identified, particularly in the *MYH2* gene.

### Protein and transcripts analyses

WB analysis revealed the presence of the full‐length N2A titin isoform, and the absence of the band at the size predicted for the p.(Arg26562Thrfs*12) allele (around 2600 kDa) in P1 (i702 in Fig. 2O). To determine whether the absence of the truncated protein was due to degradation of truncated titin mRNA by nonsense‐mediated decay (NMD), the transcript region containing exon 326 was analyzed. Reverse transcription and Sanger sequencing of cDNA revealed that the mRNA with the frameshift mutation was present, but possibly in a lower amount than the wild type allele, suggesting possible, even incomplete NMD (Fig. 2N). Coomassie blue gel staining showed loss of the band corresponding to the fast MyHC isoforms, confirmed by WB with the fast MyHC2A and 2X antibody (Fig. 2O). WB analysis of MyHC2 expression in the parents’ muscle biopsies did not show any alteration.

## Discussion

We describe here two siblings with multicore congenital myopathy and cardiac rhythm disturbances probably due to the combination of three *TTN* variants and the subsequent deficiency of fast MyHC isoforms. The clinical phenotype was similar to that of congenital titinopathies^3^ with associated hyperlaxity and hyperkeratosis in P1, and prominent amyotrophy in P2. Of note, skin alterations have never been associated with titinopathies. The MRI pattern was that of a congenital titinopathy^3^ with the addition of a very important bright signal in the upper part of the semi‐tendinous muscles in P2. Cardiac rhythm disturbances were observed in both patients, but were less severe compared with the previous description in multiminicore titinopathies.^4^ Muscle biopsies revealed pathognomonic ultrastructural features of congenital titinopathies,^5^ especially core lesions.

Three *TTN* variants, a frameshift mutation in the A band inherited from the mother and two missense mutations inherited from the father, were identified in both patients. In silico analyses suggested that the two missense mutations had deleterious consequences, based on their absence in the general population and the TITINdb predictions. In the study by Savarese et al.,^10^ homology structural modeling analyses suggested deleterious consequence of missense variants.^10^ The substitution p.(Thr6324Pro) is located on the external surface of a β strand in an Ig‐domain of the I‐band region, probably affecting the protein stability.^10^ The missense variant p.(Thr31339Ala), located in an Fn3 domain within the A‐band, could affect hydrogen bonding with other molecules and may influence the interaction with titin ligands.^10^ Moreover, Thr31339 is in a myosin‐binding domain,^17^ and the missense variant could alter titin‐myosin interactions. The effect of the two missense variants might not be quantitative but qualitative, resulting from the combination of lower stability, due to the p.(Thr6324Pro) variant, and decreased interaction with myosin, due to the p.(Thr31339Ala) variant on the same allele*.*

The p.(Arg26562Thrfs*12) variant is located in the A band where heterozygous truncating mutations represent the most common cause of dilated cardiomyopathy,^18^, ^19^, ^20^ with a dominant‐negative effect.^21^ The absence of skeletal muscle and cardiac signs of disease in the patients’ mother, who is heterozygous for this frameshift variant, does not support a dominant effect of this mutation. WB analysis did not detect any truncated titin form corresponding to the frameshift allele, suggesting a loss of function effect due to mRNA or protein degradation of the allele harboring the frameshift variant. The RT‐PCR results do not allow excluding NMD, but its effect would be limited because transcripts bearing the frameshift mutation could be detected. Alternatively, titin might be produced normally, but then it might be rapidly degraded. Our study adds new data to the few previous reports suggesting post‐translational degradation of titin.^3^, ^20^, ^22^

Interestingly, fast MyHC isoforms expression was almost completely absent (both by immunofluorescence and WB). Electron microscopy showed that some fibers harbored focal lesions with complete loss of the sarcomeric structure and of thick filaments, as previously observed.^5^ In addition to the truncated maternal allele leading to titin protein loss, the missense paternal variants probably affect titin stability (p.(Thr6324Pro)) and titin‐myosin interactions (p.(Thr31339Ala)). Consequently, these three variants might disturb the stability of titin‐myosin interactions and lead to a secondary fast MyHC isoforms defect. This is reminiscent of calpain 3 loss in patients with *TTN* variants that affect the titin‐calpain 3 interaction domain.^7^ A secondary fast MyHC defect was never reported in titinopathies, and could explain the absence of type 2A muscle fibers. The MyHC2A protein, encoded by the *MYH2* gene, is expressed in human fetal muscle and adult type 2A muscle fibers. It is one of the myosin isoforms important for mammalian skeletal muscle development.^23^ *MYH2* mutations are associated with autosomal dominant or recessive congenital myopathies. Moreover, homozygous or compound heterozygous truncating *MYH2* mutations cause recessive myopathy with ophthalmoplegia, mild‐to‐moderate muscle weakness, complete lack of type 2A muscle fibers and reduced or absent expression of the corresponding MyHC2 protein.^24^, ^25^ Except for ophthalmoplegia, P1 had a similar phenotype, particularly the lack of type 2A muscle fibers that could be due to fast MyHC protein loss.

In conclusion, our findings suggest that in these siblings, titinopathy could be caused by a titin defect associated with secondary loss of fast MyHC isoforms. Our study highlights the importance of associating a thorough phenotypical review with genetic, transcriptional and protein analyses to evaluate *TTN* variants pathogenicity. Even in the presence of missense variants, WB analyses can be useful to identify secondary deficits of proteins involved in interactions with titin and provide more arguments for evaluating *TTN* variant pathogenicity. This will also help unravel the physiopathological mechanisms of titinopathies. Our study opens the way to myosin studies in a larger number of patients with suspected titinopathy and for functional titin‐myosin interaction studies.

## Author Contributions

A.P., E.M., and M.C. conceived and designed the study; A.P., C.M., M.V., R.‐Y.C., E.P., R.J.M., T.S., I.R., P.R., N.B.R., H.G., M.K., E.M. acquired and analyzed the data; A.P., E.M., M.C., wrote the manuscript.

## Conflict of Interest

None declared.

## Supporting information


**Table S1.** List of the 54 genes included in the specific custom‐designed panel.

## References

[1] Chauveau C , Rowell J , Ferreiro A . A rising titan: TTN review and mutation update. Hum Mutat 2014;35:1046–1059. 24980681 10.1002/humu.22611

[2] Savarese M , Sarparanta J , Vihola A , et al. Increasing role of titin mutations in neuromuscular disorders. J Neuromuscul Dis 2016;3:293–308. 27854229 10.3233/JND-160158 PMC5123623

[3] Oates EC , Jones KJ , Donkervoort S , et al. Congenital titinopathy: comprehensive characterization and pathogenic insights. Ann Neurol 2018;83:1105–1124. 29691892 10.1002/ana.25241 PMC6105519

[4] Chauveau C , Bonnemann CG , Julien C , et al. Recessive TTN truncating mutations define novel forms of core myopathy with heart disease. Hum Mol Genet 2014;23:980–991. 24105469 10.1093/hmg/ddt494 PMC3954110

[5] Ávila‐Polo R , Malfatti E , Lornage X , et al. Loss of sarcomeric scaffolding as a common baseline histopathologic lesion in titin‐related myopathies. J Neuropathol Exp Neurol 2018;77:1101–1114. 30365001 10.1093/jnen/nly095

[6] Kontrogianni‐Konstantopoulos A , Ackermann MA , Bowman AL , et al. Muscle giants: molecular scaffolds in sarcomerogenesis. Physiol Rev 2009;89:1217–1267. 19789381 10.1152/physrev.00017.2009 PMC3076733

[7] De Cid R , Ben Yaou R , Roudaut C , et al. A new titinopathy: childhood‐juvenile onset Emery‐Dreifuss‐like phenotype without cardiomyopathy. Neurology 2015;85:2126–2135. 26581302 10.1212/WNL.0000000000002200 PMC4691685

[8] Yauy K , Baux D , Pegeot H , et al. MoBiDiC prioritization algorithm, a free, accessible, and efficient pipeline for single‐nucleotide variant annotation and prioritization for next‐generation sequencing routine molecular diagnosis. J Mol Diagn 2018;20:465–473. 29689380 10.1016/j.jmoldx.2018.03.009

[9] Laddach A , Gautel M , Fraternali F . TITINdb‐a computational tool to assess titin's role as a disease gene. Bioinformatics 2017;33:3482–3485. 29077808 10.1093/bioinformatics/btx424 PMC5860166

[10] Savarese M , Maggi L , Vihola A , et al. Interpreting genetic variants in titin in patients with muscle disorders. JAMA Neurol 2018;75:557–565. 29435569 10.1001/jamaneurol.2017.4899 PMC5885217

[11] Malfatti E , Olivé M , Taratuto AL , et al. Skeletal muscle biopsy analysis in reducing body myopathy and other FHL1‐related disorders. J Neuropathol Exp Neurol 2013;72:833–845. 23965743 10.1097/NEN.0b013e3182a23506 PMC5210222

[12] Richard P , Ader F , Roux M , et al. Targeted panel sequencing in adult patients with left ventricular non‐compaction reveals a large genetic heterogeneity. Clin Genet 2019;95:356–367. 30471092 10.1111/cge.13484

[13] Savarese M , Jonson PH , Huovinen S , et al. The complexity of titin splicing pattern in human adult skeletal muscles. Skelet Muscle 2018;8:11. 29598826 10.1186/s13395-018-0156-z PMC5874998

[14] Hudson B , Hidalgo C , Saripalli C , Granzier H . Hyperphosphorylation of mouse cardiac titin contributes to transverse aortic constriction‐induced diastolic dysfunction. Circ Res 2011;109:858–866. 21835910 10.1161/CIRCRESAHA.111.246819 PMC3191198

[15] Evilä A , Vihola A , Sarparanta J , et al. Atypical phenotypes in titinopathies explained by second titin mutations. Ann Neurol 2014;75:230–240. 24395473 10.1002/ana.24102

[16] Zenagui R , Lacourt D , Pegeot H , et al. A reliable targeted next‐generation sequencing strategy for diagnosis of myopathies and muscular dystrophies, especially for the giant titin and nebulin genes. J. Mol Diagn 2018;20:533–549. 29792937 10.1016/j.jmoldx.2018.04.001

[17] Muhle‐Goll C , Habeck M , Cazorla O , et al. Structural and functional studies of titin's fn3 modules reveal conserved surface patterns and binding to myosin S1 ‐ a possible role in the Frank‐Starling mechanism of the heart. J Mol Biol 2001;313:431–447. 11800567 10.1006/jmbi.2001.5017

[18] Herman DS , Lam L , Taylor MRG , et al. Truncations of titin causing dilated cardiomyopathy. N Engl J Med 2012;366:619–628. 22335739 10.1056/NEJMoa1110186 PMC3660031

[19] Akinrinade O , Koskenvuo JW , Alastalo TP . Prevalence of titin truncating variants in general population. PLoS ONE 2015;10:e0145284. 26701604 10.1371/journal.pone.0145284 PMC4689403

[20] Schafer S , de Marvao A , Adami E , et al. Titin‐truncating variants affect heart function in disease cohorts and the general population. Nat Genet 2017;49:46–53. 27869827 10.1038/ng.3719 PMC5201198

[21] Roberts AM , Ware JS , Herman DS , et al. Integrated allelic, transcriptional, and phenomic dissection of the cardiac effects of titin truncations in health and disease. Sci Transl Med 2015;7:270ra6. 10.1126/scitranslmed.3010134 PMC4560092 25589632

[22] Kellermayer D , Smith JE , Granzier H . Titin mutations and muscle disease. Pflugers Arch 2019;471:673–682. 30919088 10.1007/s00424-019-02272-5 PMC6481931

[23] Schiaffino S , Rossi AC , Smerdu V , et al. Developmental myosins: expression patterns and functional significance. Skelet Muscle 2015;5:22. 26180627 10.1186/s13395-015-0046-6 PMC4502549

[24] Tajsharghi H , Oldfors A . Myosinopathies: pathology and mechanisms. Acta Neuropathol 2013;125:3–18. 22918376 10.1007/s00401-012-1024-2 PMC3535372

[25] Tajsharghi H , Hammans S , Lindberg C , et al. Recessive myosin myopathy with external ophthalmoplegia associated with MYH2 mutations. Eur J Hum Genet 2014;22:801–808. 24193343 10.1038/ejhg.2013.250 PMC4023224

