# Supplementary material for: A new congenital multicore titinopathy associated with fast myosin heavy chain deficiency
Source: Ann Clin Transl Neurol. 2020 Apr 19;7(5):846–54. doi: 10.1002/acn3.51031 (PMC7261750; doi:10.1002/acn3.51031)
Supplement: Supplementary file 1 — Table S1. List of the 54 genes included in the specific custom‐designed panel. [file ACN3-7-846-s001.docx]

**Supplemental table 1. List of the 54 genes included in the specific custom-designed panel :**

*ACTA1* (NM_001100.3)*,* *ADAMTS2* (NM_014244.4), *ANO5* (NM_213599.2), *BAG3* (NM_004281.3), *CAV3* (NM_033337.2), *COL1A1* (NM_000088.3)*, COL1A2* (NM_000089.3), *COL3A1* (NM_000090.3), *COL5A1* (NM_001278074.1), *COL5A2* (NM_000393.3), *COL5A3* (NM_015719.3), *COL6A1* (NM_001848.2), *COL6A2* (NM_001849.3)*, COL6A3* (NM_004369.3), *COL6A6* (NM_001102608.1), *COL12A1* (NM_0004370.5), *CRYAB* (NM_001885.2), *DES* (NM_001927.3), *DNAJB6* (NM_058246.3), *DNM2* (NM_001005360.2), *DYSF* (NM_003494.3), *EMD* (NM_000117.2), *FBLN5* (NM_006329.3), *FHL1* (NM_001159702.2), *FKBP14* (NM_017946.3), *FKRP* (NM_24301.4), *FLNC* (NM_001458.4), *GAA* (NM_000152.3), *GNE* (NM_001128227.3), *HSPG2* (NM_001291860.1, NM_005529.5), *ITGA7* (NM_001144996.1), *LAMA2* (NM_000426.3), *LMNA* (NM_170707.2), *MATR3* (NM_199189.2), *MFN2* (NM_014874.3), *MYH2* (NM_017534.5), *MYH7* (NM_000257.2),*MYOT* (NM_006790.2), *NEB* (NM_001164507.1), *PLOD1* (NM_000302.3), *PNPLA2* (NM_020376.3), *RYR1* (NM_000540.2), *SEPN1* (NM_020451.2)*, SQSTM1*(NM_003900.4), *STIM1* (NM_001277961.1), *TCAP* (NM_003673.3), *TIA1* (NM_022173.2), *TNXB* (NM_019105.6), *TRIM32* (NM_012210.3), *TTN* (NM_001267550.1), *VCP* (NM_007126.3), *ZASP/LDB3* (NM_001080114.1, NM_007078.2), *VCP* (NM_007126.3), *ZASP/LDB3* (NM_001080114.1, NM_007078.2).
